# Supplementary material for: Lipid Polymorphism of the Subchloroplast—Granum and Stroma Thylakoid Membrane—Particles. I. 31P-NMR Spectroscopy
Source: Cells. 2021 Sep 8;10(9):2354. doi: 10.3390/cells10092354 (PMC8470346; doi:10.3390/cells10092354)
Supplement: Supplementary file 1 [file cells-10-02354-s001.zip › cells-1284603-supplementary.pdf]

Supplementary Information

Lipid Polymorphism of the Subchloroplast – Granum and Stroma Thylakoid Membrane – Particles. I. ^{31}P-NMR Spectroscopy

Ondřej Dlouhý[1§], Uroš Javornik[2§], Otto Zsiros[3], Primož Šket[2,4], Václav Karlický[1,5], Vladimír Špunda[1,5], Janez Plavec[2,4,6], Győző Garab[1,3*]

[1]Faculty of Science, University of Ostrava, Ostrava, Czech Republic

[2]Slovenian NMR Center, National Institute of Chemistry, Ljubljana, Slovenia

[3]Biological Research Centre, Eötvös Loránd Research Network, Szeged, Hungary

[4]EN-FIST Center of Excellence, Ljubljana, Slovenia

[5]Global Change Research Institute, Czech Acad. Sci., Brno, Czech Republic

[6]Faculty of Chemistry and Chemical Technology, Ljubljana, Slovenia

[*]Corresponding author: garab.gyozo@brc.hu

[§]These two authors contributed equally to this paper

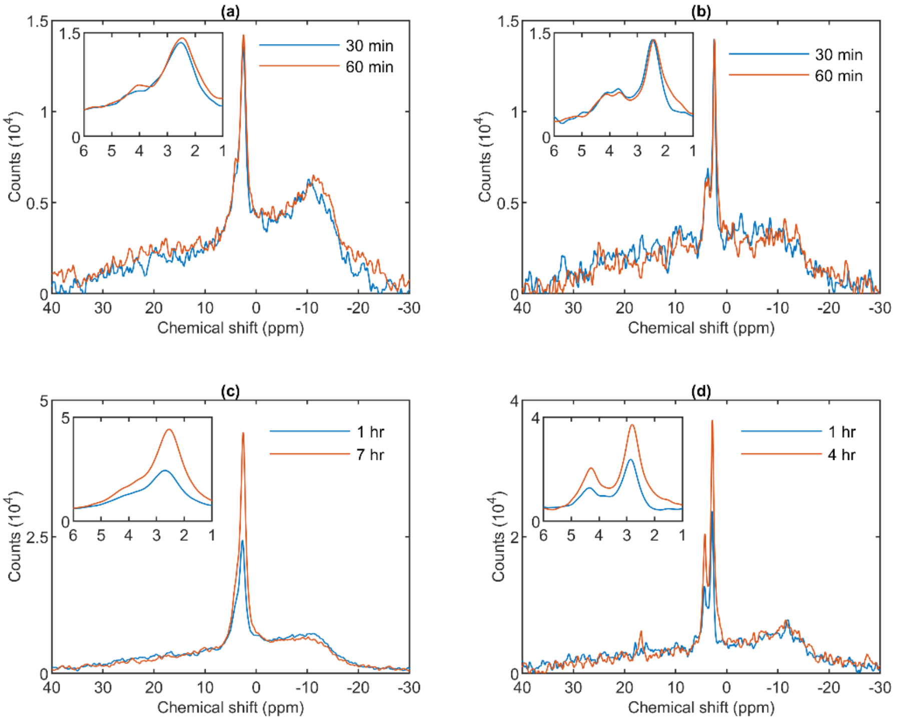

Supplementary Figure 1: Short-term (a, b) and medium-term (c, d) lipid-phase stability of granum (a, c) and stroma (b, d) thylakoid membranes at 5 °C – as reflected by the temporal variations of the ^{31}P-NMR spectra recorded at different intervals after suspending the sample. Acquisition times, 30 min (a, b) and 1 h (c, d). The insets show the isotropic regions.

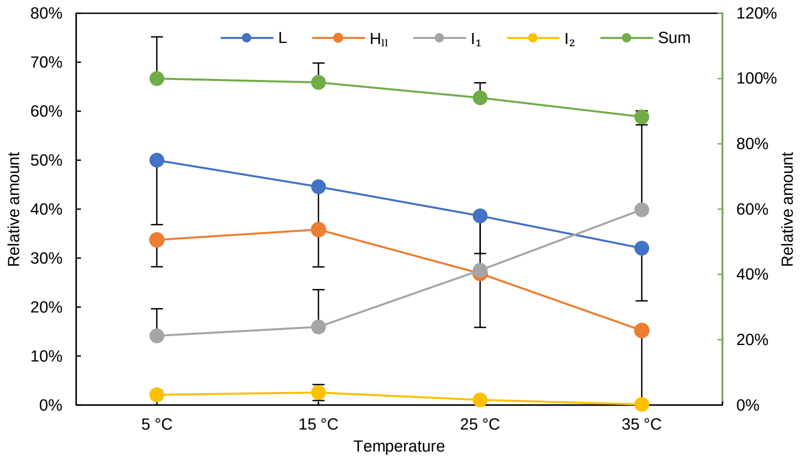

Supplementary Figure 2: Temperature dependences of the integrated areas of the ^{31}P-NMR spectral components associated with the L, H_{II}, I_1 and I_2 lipid phases (left axis) of granum TMs, and the total area (Sum) of the corresponding spectra (right axis), relative to (% of) the initial areas at 5 °C. Mean and SD values obtained from 4 experiments on 3 batches.

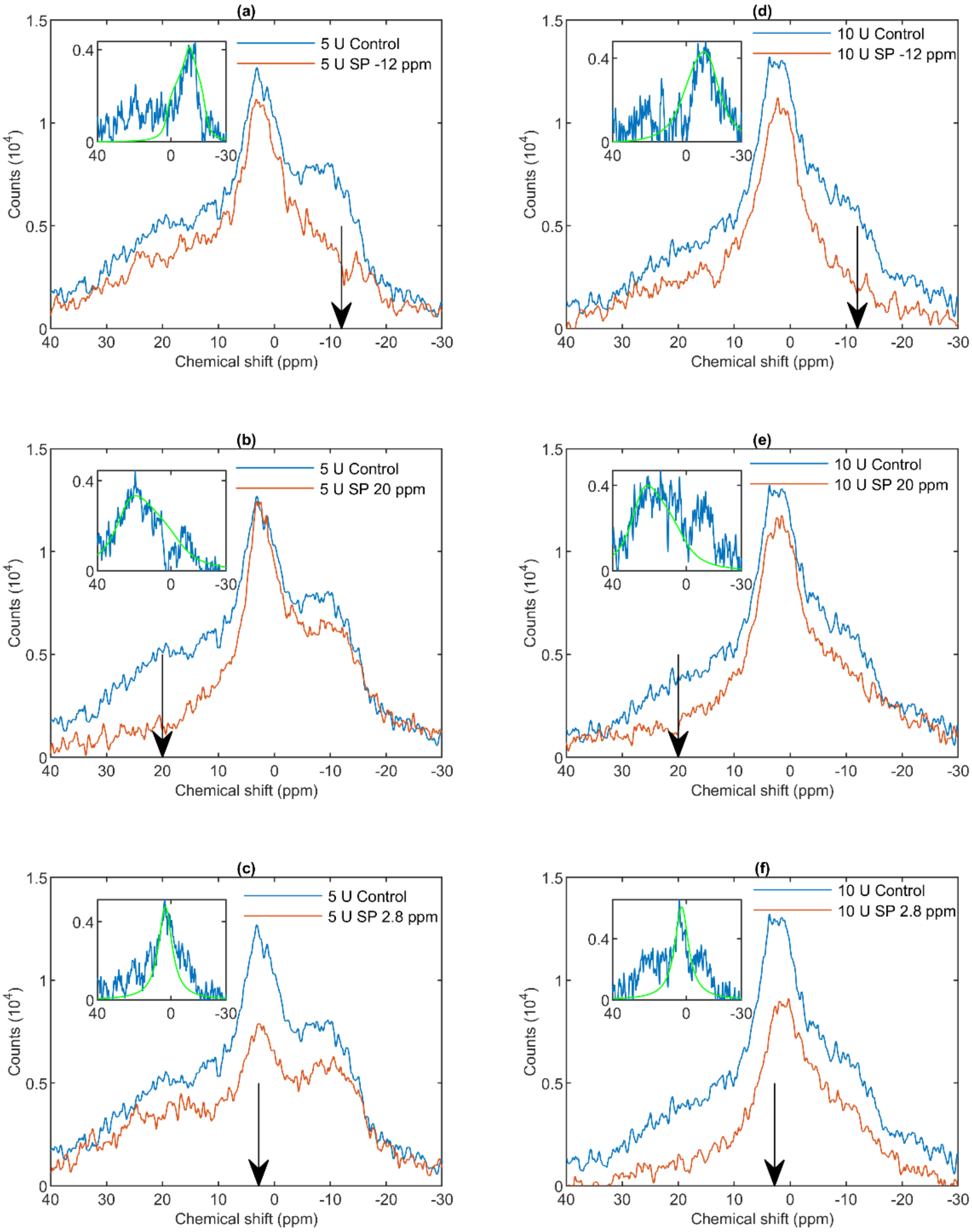

Supplementary Figure 3: ^{31}P-NMR spectra of wheat-germ treated granum thylakoid membranes in the absence (blue curves) and presence (red curves) of saturation pulses (SPs) applied at different frequencies, as indicated by the arrows, at or close to the peak position of different phases: L, -12 ppm (a, d); H_{II}, 20 ppm (b, e) and the remaining isotropic phase, I, 2.8 ppm (c, f). Wheat-germ lipase treatments: 5 U (a-c) and 10 U (d-f). Insets show the difference spectra obtained from the corresponding spectra without and with the saturation pulses; and the fitted component spectra (smooth lines); temperature, 5 °C.
